# Hybrid watermilfoil lineages are more invasive and less sensitive to a commonly used herbicide than their exotic parent (Eurasian watermilfoil)

**DOI:** 10.1111/eva.12027

**Published:** 2012-11-16

**Authors:** Elizabeth A LaRue, Matthew P Zuellig, Michael D Netherland, Mark A Heilman, Ryan A Thum

**Affiliations:** 1 Annis Water Resources Institute, Grand Valley State UniversityMuskegon, MI, USA; 2 Center for Aquatic and Invasive Plants, US Army Engineer Research and Development CenterGainesville, FL, USA; 3 SePRO CorporationCarmel, IN, USA; 4 Department of Biological Sciences, Purdue UniversityWest Lafayette, IN, USA; 5 Department of Genetics, University of GeorgiaAthens, GA, USA

**Keywords:** biological invasion, contemporary evolution, herbicide resistance, heterosis, hybridization, *Myriophyllum spicatum*

## Abstract

Hybridization may stimulate the evolution of invasiveness in human-impacted habitats if unique hybrid genotypes have higher fitness than parental genotypes. Human efforts to control invasive taxa frequently involve the intentional alteration of habitats, but few studies have considered whether hybridization can result in decreased sensitivity to control measures. Here, we investigate whether interspecific hybrids between introduced Eurasian watermilfoil (*Myriophyllum spicatum*) and native northern watermilfoil (*M. sibiricum*) are more invasive than parental Eurasian watermilfoil, especially in regard to their relative responses to an herbicide commonly applied for their control (2,4-dichlorophenoxyacetic acid; 2,4-D). In two separate laboratory experiments, hybrids on average grew faster and were less sensitive to 2,4-D compared with parental Eurasian watermilfoil. These two invasive traits appear to be common in hybrid watermilfoils, as opposed to being restricted to a few unique lineages, because they were found in a diversity of hybrid genotypes from several independent hybridization events. In addition, we found that hybrids occurred more frequently than parental species in natural lakes previously treated with 2,4-D. Our results provide compelling empirical evidence that hybridization is associated with the evolution of increased invasiveness in watermilfoils, and have important implications for their management.

## Introduction

Hybridization can stimulate the evolution of invasiveness, whereby a hybrid lineage either replaces one or both parental species or establishes in a new environment not inhabited by either parental species (Ellstrand and Schierenbeck 2000; Schierenbeck and Ellstrand 2009). This may occur through several mechanisms including heterosis (hybrid vigor), increased genetic variation/novelty, and dumping of genetic load (Ellstrand and Schierenbeck 2000; Rieseberg et al. 2007). Human activities can accelerate the evolution of invasiveness via hybridization by increasing the frequency in which previously isolated lineages come into contact and/or by creating novel environments that unique hybrid genotypes may be better suited to than parental species (Anderson and Stebbins 1954; Ellstrand and Schierenbeck 2000; Arnold and Martin 2010). In this study, we consider the case where human efforts to control invasive taxa may facilitate the evolution of invasiveness via hybridization.

Human efforts to eradicate or reduce the growth and spread of invasive taxa frequently involve the intentional alteration of habitats to create novel, stressful conditions for the target taxa. For example, application of herbicides to kill or limit invasive plant growth undoubtedly creates novel and extreme environmental conditions. In cases where populations evolve reduced sensitivity to control efforts (e.g., herbicide), the derived populations could be considered to exhibit increased invasiveness because of their increased ability to persist in the altered environment relative to populations exhibiting wild-type sensitivity to control efforts. Given the numerous traits that can be affected by hybridization, it is possible that hybridization could generate genotypes that are better suited to deal with the novel and stressful habitats created by human control efforts in comparison with parental taxa and thus facilitate the evolution of increased invasiveness in terms of displacing parental species or occurring in habitats where parental species cannot. Such increased invasiveness would obviously be of utmost management concern.

Eurasian watermilfoil (*Myriophyllum spicatum* L.; EWM) is a widespread invasive aquatic plant species in North America. EWM has hybridized with its native sister species, northern watermilfoil (*Myriophyllum sibiricum* Komarov; NWM), and many populations originally identified as invasive EWM are actually composed of these interspecific hybrids (Moody and Les 2002, 2007; Sturtevant et al. 2009; Authors in press). Moody and Les (2002) noted that *M. spicatum* × *M. sibiricum* hybrid (hereafter ‘hybrids’) populations in Connecticut, USA, displayed vegetative vigor that could indicate more aggressive growth by hybrid versus parental genotypes, although no quantitative comparison was conducted. Although native NWM is rarely considered a nuisance or targeted for control with herbicides, both EWM and hybrids are considered invasive and are frequently targeted for control with herbicides to limit their negative impacts on biodiversity and ecosystem services in many lakes and rivers. For the most part, herbicides have provided an effective means of selectively controlling EWM with minimal impact on native species (Aiken et al. 1979; Parsons et al. 2001; Madsen et al. 2002; Poovey et al. 2004). However, in recent years, there have been anecdotal reports by lake managers and residents of herbicide applications that failed to achieve the expected levels of control. In some cases, reduced control efficacy was correlated with marked morphological changes between standing watermilfoil populations (i.e., those that did not respond sufficiently to herbicide treatment) versus the earlier populations (i.e., those that responded normally to herbicide treatment). These perceived changes in morphology and herbicide response have sparked curiosity as to whether some or all hybrids exhibit reduced herbicide sensitivity. Thus, while there has been speculation among lake managers as to whether hybrids are more invasive than EWM – in terms of more aggressive vegetative growth and/or decreased sensitivity to herbicides – quantitative comparisons between hybrid and parental EWM have not been conducted. Indeed, if hybrids do grow faster and are less herbicide sensitive, then new management practices need to be developed for better control.

In this study, we ask whether hybrids are more invasive than parental EWM in regard to two potentially important aspects of invasiveness: vegetative growth and herbicide sensitivity. We focus specifically on the comparison between EWM and hybrids because NWM is not of management concern. Although several different aquatic herbicides are used to control watermilfoils, we focused our study on the most widely used herbicide for watermilfoil control – the synthetic auxin herbicide 2,4-dichlorophenoxyacetic acid (2,4-D). We used a laboratory assay to compare the growth of hybrid versus EWM genotypes at different concentrations of 2,4-D. Because watermilfoils can reproduce asexually by vegetative fragmentation (Aiken et al. 1979), it is possible that any watermilfoil genotype(s) exhibiting reduced 2,4-D sensitivity could spread to different lakes via asexual propagation. If true, we might find the same clonal genotype(s) in different lakes exhibiting reduced sensitivity. Alternatively, if reduced sensitivity independently arises, we expect to find reduced sensitivity in different populations consisting of different clonal genotypes. Thus, we sampled populations that were found to be genetically distinct in this and an earlier study (Zuellig and Thum 2012). Specifically, we included genetically diverse hybrids from different hybridization events in our experiments to test whether reduced 2,4-D sensitivity is causally associated with hybridity versus being restricted to one or a small number of unique genotypes. Finally, we analyzed distribution patterns of hybrid and parental watermilfoil genotypes in lakes that have versus have not been treated with 2,4-D to determine whether hybrids are associated with lakes having a history of 2,4-D management.

## Materials and methods

### Study populations and laboratory cultures

The laboratory 2,4-D sensitivity data were collected in two separate experiments, each of which included genotypes from different EWM and hybrid populations. In the first experiment, we collected watermilfoils from lakes in the Menominee River watershed in Michigan's Upper Peninsula and adjacent Wisconsin, USA (four EWM and six hybrid populations; Table S1). We focused the first experiment on the Menominee River watershed for two reasons. First, verbal reports from lake managers and residents identified two populations of suspected hybrids that exhibited reduced responses to field applications of 2,4-D. Second, 2,4-D is the only herbicide that has been used to control nuisance watermilfoils in the Menominee River watershed, whereas several other herbicides are routinely used in addition to 2,4-D in most other regions of the USA. Thus, the Menominee River watershed provided a unique opportunity to study the relative 2,4-D sensitivities of hybrids and EWM without any potentially confounding effects of management with other herbicides. For this same reason, we also studied the distribution patterns of hybrid versus parental watermilfoils in the Menominee River watershed (see section). However, genetic diversity in the Menominee River watershed represents a small subset of the genetic variation in hybrid and EWM populations. For example, at least two genetically distinct lineages of EWM have been introduced to North America (Zuellig and Thum 2012), and only one of these two EWM lineages is found in the Menominee River watershed (Table S1). Similarly, hybrids as a group are genetically diverse and have arisen independently through many distinct hybridization events among distinct parental populations (Zuellig and Thum 2012; Table S1). Thus, in our second experiment, we collected genetically distinct populations of watermilfoils from throughout the Lower Peninsula of Michigan, USA (six hybrid and nine EWM populations; Table S2). Our study focused on whether hybrids exhibit traits that make them more invasive than their invasive parent in managed habitats; thus, we did not include native NWM in this study because it is not considered a nuisance species and is not targeted for treatment with herbicides. By including a diverse set of populations and genotypes of EWM and hybrids in the two experiments, we were able to evaluate whether reduced 2,4-D sensitivity has arisen in one or a small number of unique hybrid genotypes that have spread through asexual reproduction, or whether hybridization is repeatedly associated with reduced 2,4-D sensitivity across distinct lineages.

Hybrid and EWM were sampled from wild populations in 2011 and used to establish cultures as a laboratory source of plants for our 2,4-D assay experiments. We planted 50 or more apical meristems (∼15 cm) from randomly collected plants in each lake into 18.9-L buckets containing potting soil supplemented with 2.2 mL/kg Osmocote (19:6:12, nitrogen/phosphorus/potassium). We planted two buckets in the aforementioned manner for each lake, and the buckets were arbitrarily allocated to eight 1136-L tanks at the Annis Water Resources Institute. Each tank contained a mix of EWM and hybrid populations, but populations were kept separate within each tank with a mesh netting divider, and daily maintenance was conducted to ensure no cross-contamination of different populations within the same tank. Tanks were filled with filtered water from nearby Muskegon Lake, and each was lit with a full-spectrum sodium lamp (Sylvania M1000/U M47/S Metalarc) on a 12:12 h light/dark cycle. Plants were vegetatively propagated every 2–3 weeks by cutting ∼20–30 cm of each stem and replanting; cut plants readily establish roots and rapid growth within several days under these conditions. Plants were propagated in this manner for 2–3 months before the experiment to ensure that all plants used in the assay were from new growth that was healthy and actively growing to reduce any maternal or plastic effects carried over from field conditions.

**Figure 1:**
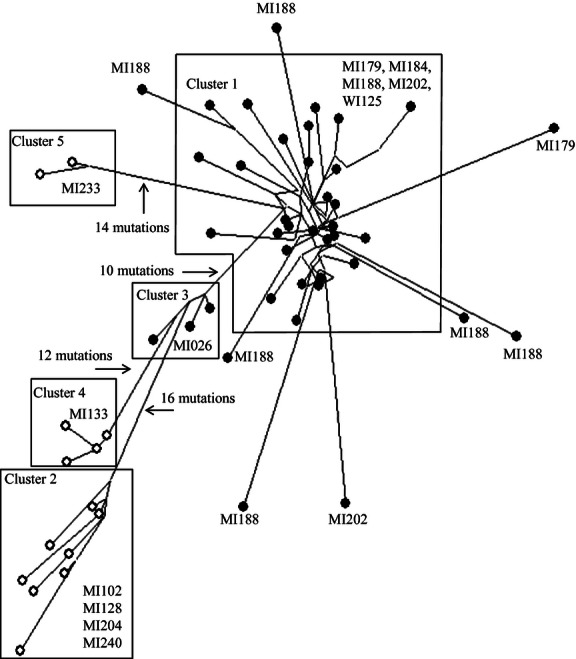
Minimum-spanning network of 54 unique hybrid genotypes (based on amplified fragment length polymorphisms) collected from 12 lakes and used to establish laboratory cultures for our 2,4-D experiments. Black circles are genotypes from Menominee River watershed lakes, and white circles are genotypes from lakes in the Lower Peninsula of MI. Boxes enclose distinct genetic clusters that indicate different hybridization events. Populations found within each cluster are labeled within boxes (same population ID used in Zuellig and Thum 2012). Lengths of lines are proportional to the number of mutations separating genotypes, and the number of mutations (band differences) separating distinct clusters is indicated with an arrow.

**Figure 2:**
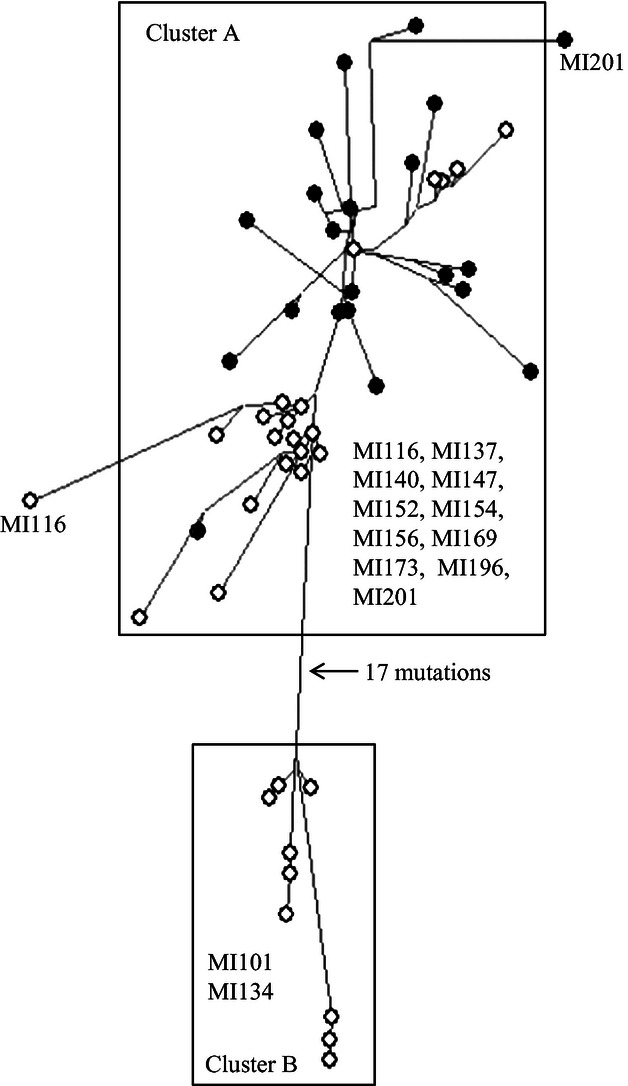
Minimum-spanning network of 51 unique Eurasian watermilfoil genotypes (based on amplified fragment length polymorphisms) collected from 13 lakes and used to establish laboratory cultures for our 2,4-D experiments. Black circles are genotypes from Menominee River watershed lakes, and white circles are genotypes from lakes in the Lower Peninsula of MI. Boxes enclose distinct genetic clusters that indicate different introduction events. Populations found within each cluster are labeled within boxes (same population ID used in Zuellig and Thum 2012). Lengths of lines are proportional to the number of mutations separating genotypes, and the number of mutations (band differences) separating distinct clusters is indicated with an arrow.

At the time of culture establishment, we arbitrarily selected ∼8–30 individuals to be genotyped with 99 amplified fragment length polymorphism markers (AFLP markers) using the methods of Zuellig and Thum (2012). We conducted the AFLP analysis for two reasons. First, we used the AFLP data, along with visual observations of the plant cultures, to ensure that each culture from each population consisted entirely of either EWM or hybrid. Second, we used these data as a means to illustrate the genetic diversity that was incorporated into our study. In total, we identified 54 unique genotypes or clones (i.e., AFLP profiles that differed by at least one band) among our 12 hybrid populations and 51 unique genotypes among our 13 EWM populations (see Tables S1 and S2 for number of individuals genotyped per population). We constructed minimum-spanning networks as a means to visually illustrate the genetic diversity of hybrid and EWM lineages included in the study populations. The networks were constructed with NETWORK 4.6.1.0 using the median-joining approach (Bandelt et al. 1999) and MP (maximum parsimony) option (Polzin and Daneschmand 2003), with AFLPs treated as binary data. We did not do a formal phylogenetic treatment of the lineages because the hybrid lineages are reticulate. Nevertheless, the hybrid genotypes can be divided into at least five different genetic groups based on relatively large numbers of mutations separating them (two of these groups correspond to those delineated in Zuellig and Thum (2012), but additional groups were identified here from populations that were not included in that study; Fig. 1). These distinct hybrid groups likely represent different hybridization events among distinct parental populations. Similarly, EWM genotypes can be divided into two clearly distinct genetic groups, and these correspond to those identified in Zuellig and Thum (2012); Fig. 2). We note that we did not keep track of individuals in the cultures because it was intractable to do so through the several rounds of vegetative propagation, and we therefore do not know which exact genotype each experimental plant was. However, because of the diversity of unique genotypes among different populations, we are certain that our experiment included a diverse set of hybrid genotypes that represent at least several independent hybridization events. Similarly, we are certain that our experiments included different EWM clones from the two genetically distinct lineages representing independent introductions (see Zuellig and Thum 2012; see also Table S2).

### 2,4-D Sensitivity assays

At the beginning of each experiment, we randomly harvested healthy, actively growing, 12-cm apical meristems from our established cultures of each population. We recorded the initial wet weight of each meristem after gently blotting it dry with a paper towel. Plants were arbitrarily assigned to a 2,4-D treatment or the control (water) and were individually labeled and wrapped in a permeable netting to allow sufficient contact with the liquid in their treatment (see below). Herbicide exposures occurred in one plastic tub per treatment containing 15 L of water (control) or 15 L of water mixed with analytical grade 2,4-D (Fisher Scientific, Pittsburgh, PA, USA). Owing to time and space constraints, we included all replicates for each treatment in a single plastic tub (i.e., one tub each for each 2,4-D concentration or control). We are aware that this logistical constraint may be interpreted as pseudo-replication of the treatments. However, we wrapped each individual separately in a permeable mesh netting to allow for each plant to experience potential microhabitat variations because of 2,4-D concentration differences or light variation throughout each tank, and we therefore considered each individual plant to be the unit of replication. In this way, our study design is similar to experiments in incubators or environmental chambers that are not easily replicated. Despite this potential design flaw, the consistent proportional decrease in growth with increasing herbicide levels across both experiments suggests that the result is real and that tank effects were minimal or nonexistent.

In the Menominee River watershed populations, we had four levels of 2,4-D concentrations (100, 150, 200, and 500 μg/L) and a control and had sample sizes of five individuals per population per treatment (except *N* = 4 at 100 μg/L for MI201 and *N* = 0 at 500 μg/L for MI154; *N*_Total_ = 244). On the basis of the qualitative response patterns in the first experiment, we reduced the number of 2,4-D levels to two (200 and 500 μg/L) and the sample sizes to four individuals per population per treatment (except *N* = 3 for MI133 at 0 μg/L and MI233 at all treatments; *N*_Total_ = 176) in the second experiment (Lower Peninsula populations) to accommodate the larger number of populations included. We confirmed that we maintained our target 2,4-D concentrations over 2 days with the RaPID Assay® 2,4-D Test Kit (SIDX, Newark, DE, USA) for water samples taken at the beginning and end of the exposure, including the control. Plants were kept in these treatments for 2 days to allow sufficient time for 2,4-D uptake, which is similar to a typical exposure time in the field (2,4-D can rapidly dilute in natural waterbodies, see below).

It is important to note that we intentionally chose 2,4-D concentrations and exposure times that are slightly below recommended target concentrations but likely lie within the ranges of what many plants experience in the field. Green and Westerdahl (1990) found that 2,4-D concentrations of 2 mg/L for 24 h, 1 mg/L for 36 h, and 0.5 mg/L for 72 h were sufficient for EWM control. Indeed, a preliminary experiment with several of our populations confirmed that 2,4-D had lethal effects on both EWM and hybrids at concentrations above 2 mg/L. However, while target concentrations may routinely be 1–2 mg/L for field treatments, many applications fail to reach this concentration. 2,4-D is most frequently applied as a ‘spot treatment’ to the specific area(s) where watermilfoils are a nuisance, as opposed to whole-lake applications at the target concentration. Recent data indicate that these treatments can rapidly dilute from the treatment site into the rest of the lake to concentrations at or below those used in our study (Bugbee et al. 2003; WIDNR and USACE of ERDC 2011). In addition, plants undoubtedly occur in many lakes outside of the treated areas, and these plants are certain to experience concentrations that are below the target concentration. Plants in these peripheral areas can serve as sources for recolonization of treated areas, and the rate of recolonization will influence the evaluation of how well the treatment worked (e.g., faster recolonization would be perceived as lower control efficacy). We therefore argue that while individual field applications may target concentrations closer to 1 or 2 mg/L that would effectively control the targeted populations, our experimental conditions simulate the lower concentrations and exposure times that many populations are likely to experience under realistic field conditions when 2,4-D rapidly dilutes and dissipates.

After the 2-day exposure, stems were individually planted in a 115-mL container filled with potting soil supplemented with 2.2 mL/kg Osmocote and capped with sand to prevent leaching of soil into the water column. Each container had three small holes at the base that plants could grow roots through. Each container was randomly placed in one of three 55-L plastic bins located in an 1136-L tank. Each plastic bin was filled with 20 L of potting soil that plants could extend their roots into from their containers, and the potting soil was capped with ∼5-cm sand. Plants were allowed to grow for 22 and 20 days in the first (Menominee River watershed populations) and second (Lower Peninsula populations) experiments, respectively. These time periods are sufficient to observe any negative effects of 2,4-D. The grow-out periods had average water temperatures of 16.5 and 17.3°C, respectively, which are representative of the temperatures that plants would experience during 2,4-D applications in our study areas (April to June, when they are typically applied). After this grow-out period, we measured gained length (final length minus 12 cm) and total gained wet weight (final wet weight minus initial wet weight).

We tested for differences in growth and 2,4-D sensitivity in hybrids and EWM using two-way nested anovas with 2,4-D concentration and source lake nested within taxon (hybrid versus EWM) as factors. We tested for the fixed effects of taxon, treatment, and their interaction, and the random effect of source lake nested within taxon. Differences in 2,4-D sensitivity are indicated by differences in the proportionate growth in a 2,4-D treatment relative to a control, and our statistical analyses used this value calculated as gained length of a 2,4-D-treated plant (Length_treated_) divided by the population mean of gained length of the untreated control (Length_control_). Thus, a value of 0.0 reflects high sensitivity, whereas 1.0 reflects no sensitivity. Significance was assessed at an α = 0.05 for all statistical analyses. We performed multiple comparisons as pairwise *t*-tests with a false discovery rate correction (Benjamini and Hochberg 1995). We used square root transformations to meet the assumption of normality. All statistical analyses were conducted in R (R Development Core Team 2011).

### Distribution of hybrid and parental watermilfoils in 2,4-D-treated versus untreated lakes

We examined distribution patterns of hybrid and parental genotypes in natural populations in the Menominee River watershed to determine whether hybrid genotypes were more abundant in lakes with a history of 2,4-D treatment. If hybrids exhibit reduced responses to 2,4-D applications, then hybrids are expected to be more common in lakes with a history of 2,4-D management.

To test this hypothesis, we selected *a priori* eight lakes in the Menominee River watershed where 2,4-D has been applied to control nuisance watermilfoils at least twice within the past 10 years and eight lakes that have watermilfoils present but have never been treated (Table S1 in 2010 and 2011). We collected 8–32 plants from each of 1–8 plant beds distributed throughout each lake. We identified samples as EWM, NWM, or hybrid using AFLPs following the procedures outlined in Zuellig and Thum (2012). To test whether hybrids were over-represented in 2,4-D-treated versus untreated lakes, we performed two different one-tailed Fisher's exact tests: ‘by lake’ and ‘by individual’. For the ‘by lake’ analysis, we used presence/absence data for each lake where the presence of a parent or hybrids was counted as ‘1′ and the absence was counted as ‘0′. Because several lakes contained both parents and hybrids, the ‘by lake’ test violates the assumption of mutual exclusivity (i.e., four lakes were included in both categories because they had both hybrid and parental genotypes). Therefore, we performed a second Fisher's exact test where we categorized each identified individual plant as being in either a treated or untreated lake (i.e., ‘by individual’). While this method violates the assumption of independent random sampling (the treated and untreated lakes are each treated as a single population), it has the advantage of taking into account the relative abundance of parents and hybrids in each lake.

## Results

**Figure 3:**
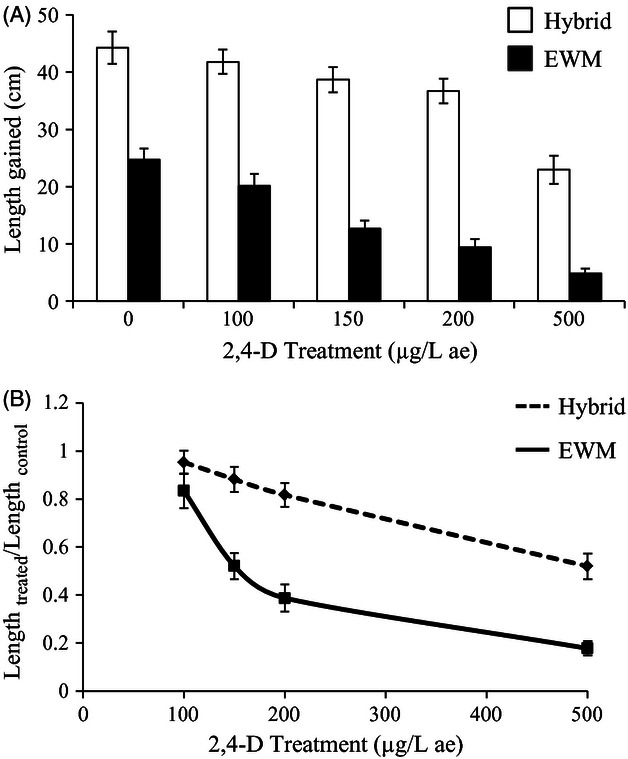
Response of hybrid and Eurasian watermilfoil (EWM) from the Menominee River watershed to four treatments of 2,4-D and a control after 22 days of growth with the mean (A) length gained (*N* = 244) and (B) length at a treatment of 2,4-D relative to length at the control (Length_treated_/Length_control_) (*N* = 194). Untransformed data are shown. Error bars are ±SEM. Trend line is included in (A) for visual interpretation. ae = acid equivalent. Statistical significance was determined with pairwise *t*-tests using a false discovery rate adjustment. Hybrids and EWM were significantly different at all treatment levels for every variable except at 100 μg/L 2,4-D in (B).

**Figure 4:**
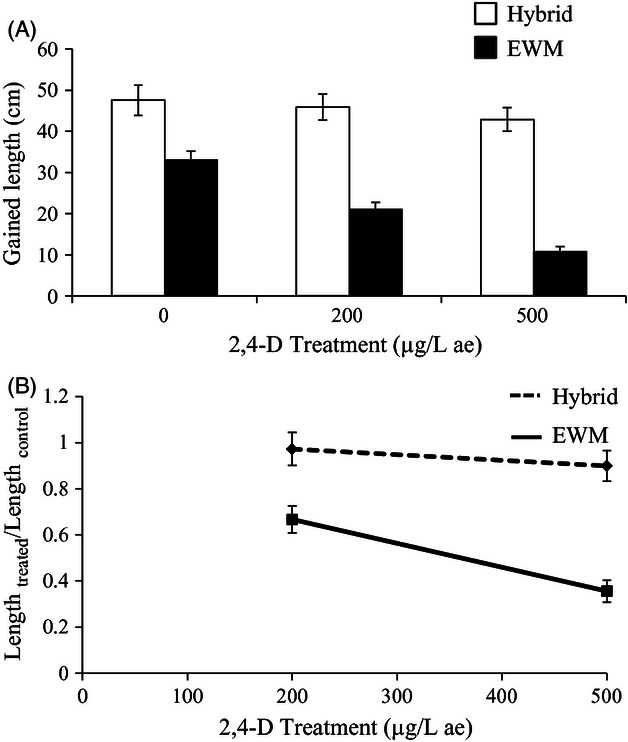
Response of hybrid and Eurasian watermilfoil (EWM) from the Lower Peninsula of Michigan, USA, to two treatments of 2,4-D and a control after 20 days of growth with mean (A) length gained (*N* = 176) and (B) length at a treatment of 2,4-D relative to length at the control (Length_treated_/Length_control_) (*N* = 118). Untransformed data are shown. Error bars are ±SEM. Trend line is included in (A) for visual interpretation. ae = acid equivalent. Statistical significance was determined with pairwise *t*-tests using a false discovery rate adjustment. Hybrids and EWM were significantly different at all treatment levels. EWM, Eurasian watermilfoil.

Hybrids had higher absolute growth; hybrids were on average longer than EWM in all treatments and the controls in both experiments (Tables 1A and 2A Figs 3A and 4A). The statistical results for gained length and wet weight were qualitatively similar and tightly correlated (Pearson's *r* = 0.88); for brevity, we only present results for gained length. Thus, hybrids grew faster than EWM regardless of whether they were treated or not.

**Table 1:** anova results for Menominee River watershed 2,4-D sensitivity experiment. (A) length gained and (B) length at a treatment of 2,4-D relative to length at the control (Length treated/Length control). Data were square root transformed. Nesting variables appear inside parentheses, and × indicates interaction terms

	df	SS	MS	*F*	*P* (>*F*)
(A) Length gained					
Taxon (EWM, Hybrid)	1	315.1	315.1	301.7	<0.001
Treatment	4	152.7	38.17	36.55	<0.001
Population (Taxon)	8	22.08	2.76	2.64	0.009
Taxon × Treatment	4	16.33	4.08	3.91	0.005
Population (Taxon) × Treatment	31	30.9	0.99	0.95	0.541
Residuals	195	203.7	1.05		
(B) Length_treated_/Length_control_					
Taxon (EWM, Hybrid)	1	2.07	2.07	71.31	<0.001
Treatment	3	3.21	1.07	36.86	<0.001
Population (Taxon)	8	0.47	0.06	2.03	0.046
Taxon × Treatment	3	0.44	0.15	5.01	0.002
Population (Taxon) × Treatment	23	0.79	0.03	1.17	0.264
Residuals	155	4.5	0.03		

SS, sum of squares; MS, mean sum of squares; EWM, Eurasian watermilfoil.

**Table 2:** anova results for Lower Peninsula 2,4-D sensitivity experiment. (A) length gained and (B) length at a treatment of 2,4-D relative to length at the control (Length treated/Length control). Data were square root transformed. Nesting variables appear inside parentheses, and × indicates interaction terms

	df	SS	MS	*F*	*P* (>*F*)
(A) Length gained					
Taxon (EWM, Hybrid)	1	210.43	210.43	168.60	<0.001
Treatment	2	80.46	40.23	32.23	<0.001
Population (Taxon)	13	33.81	2.60	2.08	0.019
Taxon × Treatment	2	32.25	16.13	12.92	<0.001
Population (Taxon) × Treatment	26	32.09	1.23	0.99	0.487
Residuals	131	163.50	1.25		
(B) Length_treated_/Length_control_					
Taxon (EWM, Hybrid)	1	2.19	2.19	71.52	<0.001
Treatment	1	0.74	0.74	24.26	<0.001
Population (Taxon)	13	1.40	0.11	3.52	<0.001
Taxon × Treatment	1	0.28	0.28	9.22	0.003
Population (Taxon) × Treatment	13	0.15	0.01	0.37	0.976
Residuals	88	2.70	0.03		

SS, sum of squares; MS, mean sum of squares; EWM, Eurasian watermilfoil.

Hybrids were also on average less sensitive to 2,4-D than EWM. In both experiments, hybrid populations had higher means for the proportion of length in 2,4-D treatments relative to controls (Tables 1B and 2B, Figs 3B and 4B). Furthermore, the effect of increasing 2,4-D concentration was lower on hybrids compared with EWM (i.e., significant taxon × treatment interaction; Tables 1B and 2B). However, the effect of increasing 2,4-D concentration was similar among different populations of hybrids and among different populations of EWM (i.e., no significant population (taxon) × treatment interaction; 1B and 2B). Thus, reduced sensitivity was common across hybrid populations, whereas no EWM populations exhibited reduced 2,4-D sensitivity (see Tables S3 and S4 for population means).

In our study of the distribution of hybrid and parental watermilfoils in 2,4-D-treated versus untreated lakes, we found that hybrids occurred more frequently in 2,4-D-treated lakes in the Menominee River watershed (Table S1). The statistical significance of this pattern held whether we used ‘by lake’ or ‘by individual’ Fisher's exact tests (*P* = 0.0359 and *P* < 0.0001, respectively; see Materials and Methods for details). Distribution patterns for 2010 and 2011 were qualitatively the same (only 2010 shown, Table S1).

## Discussion

Our study provides compelling evidence that interspecific hybrid lineages between introduced EWM and native NWM are more invasive than pure parental EWM, especially in novel habitats resulting from the application of the herbicide 2,4-D, which is routinely used to control nuisance populations of watermilfoil. Specifically, we have shown that hybrid watermilfoil genotypes exhibited faster vegetative growth and reduced sensitivity to 2,4-D in two laboratory experiments, and that they occurred more frequently than parental watermilfoil species in lakes with a history of 2,4-D treatment. Furthermore, our comparison of multiple, genetically distinct hybrid and EWM demonstrates that increased vegetative growth and reduced 2,4-D sensitivity are generally associated with hybridity in invasive watermilfoils. These traits are not restricted to one or a small number of closely related hybrid genotypes that have extensively spread among water bodies via asexual propagation, but instead appear in multiple, independently derived hybrid lineages, suggesting that hybridization predictably leads to increased invasiveness in natural populations (though the genetic mechanism(s) are currently unknown).

Here, we follow Ellstrand and Schierenbeck's (2000) definition for the evolution of invasiveness via hybridization whereby a hybrid lineage either replaces one or both parental species or becomes established in a habitat not previously inhabited by either parent species. In our case, parental EWM is itself an invasive species, so the appearance of invasiveness *per se* has not arisen solely from hybridization. However, our results demonstrate that hybrids on average have two traits – increased vegetative vigor and decreased 2,4-D sensitivity – that make them relatively more invasive than pure parental EWM lineages from which they partially derive. In particular, our laboratory experiments predict that hybrids are more likely than EWM to persist in lakes that have been treated with 2,4-D. Indeed, hybrids did occur more frequently in 2,4-D-treated lakes compared with parental species in the Menominee River watershed, where 2,4-D is the only aquatic herbicide that has been used for the control of nuisance watermilfoils. Unfortunately, a lack of historical records of NWM, EWM, and hybrids makes it impossible to determine from current distributions alone whether hybrids have displaced parental watermilfoils in 2,4-D-treated lakes or whether the pattern arose from a different mechanism such as more frequent targeting of hybrid populations for treatment or higher colonization of lakes with human activities by hybrids.

The higher vegetative growth rate of hybrids in our experimental controls suggests that hybrids could have a competitive advantage over – and ultimately displace – parental species even in untreated lakes. It is unclear why hybrids were not commonly found in untreated lakes in the Menominee River watershed whereas parental species were. It is possible that hybrid lineages will eventually take over these lakes. Or, it is possible that there are unidentified fitness trade-offs between hybrids and parental species in 2,4-D-treated versus untreated lakes. Finally, it is possible that management activities such as 2,4-D treatments accelerate a process of displacement if parental species exhibit priority effects that suppress the initial establishment of hybrid genotypes in the absence of management activities. These alternative hypotheses could be tested through field reciprocal transplant experiments and pre- versus post-treatment genetic monitoring of all future lakes where herbicide management regimes are initiated. However, such studies were beyond the scope of this one, which focused on using controlled experiments to test the hypothesis that hybrids exhibit reduced 2,4-D sensitivity relative to invasive parental EWM.

Three aspects of our study warrant further discussion. First, in both experiments, we conducted the 2,4-D exposures for each treatment in a single plastic tub as opposed to using replicate tubs as experimental blocks. We recognize that the latter statistical design would have been better to guard against potential pseudo-replication. However, logistical constraints at the time of our experiments precluded us from doing this. Nevertheless, we argue that the clear decreases in growth with increasing 2,4-D concentrations along with the qualitatively similar results in the two independent experiments strongly suggest that our results represent bona fide responses to 2,4-D as opposed to spurious results from pseudo-replication.

Second, because we used plants collected from the wild in our experiments, it is possible that phenotypic plasticity exhibited in the field carried over to our experiments. While we cannot rule this out, we find plasticity unlikely to have qualitatively impacted our results and interpretations for the following reasons: (i) We propagated all experimental plants through several cuttings and replantings for 2–3 months. Therefore, our experimental populations had a long time to adjust growth and physiology to the laboratory conditions. (ii) We collected all plants early in the growing season before any 2,4-D treatments had been applied, and thus, growth characteristics could not be explained by any carryover plasticity from recent exposure to 2,4-D. However, as both hybrids and EWM can be perennial and reproduce via asexual reproduction (Aiken et al. 1979), it is possible that any given genet could have been exposed to 2,4-D at some point in previous season(s). (iii) Nevertheless, differences in 2,4-D exposure history cannot solely explain the qualitative differences between hybrid versus pure EWM lineages because all nine of the EWM populations included in the second experiment have been treated many times before with 2,4-D. Thus, if phenotypic plasticity is important in explaining our experimental results, it is manifest as differences in the degree of plasticity between hybrids and EWM as opposed to carryover effects from previous exposures. That being said, common garden experiment(s) using artificially generated hybrid lineages and conducted over multiple generations should be conducted in the future to rule out the potential effects of phenotypic plasticity in wild-caught plants.

Finally, our study design does not allow us to infer the evolutionary genetic mechanism(s) for why hybrids exhibit increased vegetative growth and reduced 2,4-D sensitivity relative to EWM. Heterosis is often manifested as higher growth rates and metabolism, and decreased sensitivity to stress (Goff 2011), and it is therefore possible that faster growth and reduced 2,4-D sensitivity results from heterosis in first (or early)-generation interspecific hybrids. Alternatively, it is possible that hybrid populations have an increased ability to respond to selection owing to greater genetic variation. For example, hybrid populations may combine alleles from EWM that make them weedy and invasive with locally adapted native alleles. In fact, because we did not include NWM in our study, it is possible that hybrids exhibit intermediate trait values for growth rate or 2,4-D sensitivity. However, we find this unlikely because NWM is not considered a nuisance species. Experimental studies comparing artificially generated hybrids of different genotypes to parental genotypes over multiple generations should shed light on the underlying genetic control of hybrid invasiveness.

### Management implications

Our findings have important implications for the management of invasive watermilfoil populations. Specifically, they demonstrate that invasive hybrid watermilfoils are less likely to be inhibited by management with 2,4-D in comparison with parental EWM. Furthermore, the decreased sensitivity to 2,4-D does not appear to be restricted to one or a small number of lineages, but rather appears to be a common phenomenon across different hybrid lineages. However, there is still much to be learned about how natural populations of hybrids respond to operational 2,4-D treatments in the field, and our study identifies two specific types of data that should be immediately incorporated into field studies or routine monitoring of 2,4-D treatments: (i) genetic data to distinguish hybrids from parental species and (ii) 2,4-D concentration and exposure times in operational treatments. We briefly discuss these two aspects in turn below.

Hybrids are difficult to distinguish from parental watermilfoils on the basis of morphology alone, and genetic analyses are required for accurate identifications (Moody and Les 2007). Managers, consultants, and regulators are increasingly utilizing genetic methods to confirm suspected populations of hybrids, but many lake managers do not. Furthermore, quantitative monitoring of plant distribution and abundance pre- versus post-treatment are not routinely conducted or required. Thus, there are very few quantitative data available to determine whether there are any general patterns in the qualitative responses of hybrid versus parental watermilfoils to operational 2,4-D treatments, as well as whether there are any predictable shifts in the relative abundance of parental versus hybrid watermilfoils pre- versus post-treatment.

Surprisingly, despite its widespread use, 2,4-D concentrations and exposure times are rarely measured in the field, and thus, quantitative data for comparing the actual responses of hybrid versus parental watermilfoils to operational 2,4-D treatments are lacking. Our laboratory experiment used 2,4-D concentrations that are lower than the recommended target concentrations of 1–2 mg/L for the successful control of EWM (Green and Westerdahl 1990), because recent studies demonstrate that 2,4-D can dilute and dissipate from target concentrations to those within the range of our experiments in natural settings (see Materials and Methods for details; Bugbee et al. 2003; WDNR and USACE ERDC 2011). Thus, we believe that our experimental conditions are representative of many operational 2,4-D field applications. However, field data on the actual concentrations and exposure times, in combination with quantitative responses of accurately identified hybrid versus parental watermilfoils, are critical for determining best management practices for hybrid watermilfoils.

## Data archiving

Data for this study is available in the supporting information.

## Supporting Information

Data S1 Raw AFLP data for Figs 1 and 2.

Data S2 Raw data from 2,4-D sensitivity assay.

Table S1 Populations from the Menominee River Watershed, Michigan and Wisconsin, USA used in the 2,4-D sensitivity assays and natural distribution patterns analysis.

Table S2 Populations from the Lower Peninsula of Michigan, USA used in the 2,4-D sensitivity assays.

Table S3 Responses of individual hybrid and Eurasian watermilfoil (EWM) populations from the Menominee River watershed to four treatments of 2,4-D and a control after 22 days of growth with (a) growth at a treatment of 2,4-D relative to growth at the control (Length treated/Length control), and (b) length gained.

Table S4 Responses of individual hybrid and Eurasian watermilfoil (EWM) populations from the Lower Peninsula of MI, USA to two treatments of 2,4-D and a control after 20 days of growth with (a) growth at a treatment of 2,4-D relative to growth at the control (Length treated/Length control), and (b) length gained (cm).
