# Supplementary material for: Hybrid watermilfoil lineages are more invasive and less sensitive to a commonly used herbicide than their exotic parent (Eurasian watermilfoil)
Source: Evol Appl. 2012 Nov 16;6(3):462–71. doi: 10.1111/eva.12027 (PMC3673474; doi:10.1111/eva.12027)
Supplement: Table S1 — Populations from the Menominee River Watershed, Michigan and Wisconsin, USA used in the 2,4-D sensitivity assays and natural distribution patterns analysis. [file eva0006-0462-sd3.pdf]

Table S1. Populations from the Menominee River Watershed, Michigan and Wisconsin, USA used in the 2,4-D sensitivity assays and natural distribution patterns analysis. Population identification, 2,4-D treatment history, biotype identification used in 2,4-D sensitivity assays, the genetic cluster from AFLPs in the minimum-spanning network (see Fig.'s 1 and 2), and number of each taxon identified in 2010 and 2011 with AFLPs for the natural distribution patterns study. EWM = Eurasian watermilfoil, NWM = northern watermilfoil

				2010			2011		
Population	Treatment Category	2,4-D Assay Biotype ID	Genetic Cluster	EWM	NWM	Hybrid	EWM	NWM	Hybrid
MI026	Treated	Hybrid	3			25			24
MI202	Treated	Hybrid	1			26			20
WI125	Treated	Hybrid	1			24			23
MI154	Treated	EWM	A	17	15		22		
MI179	Treated	Hybrid	1			24			16
MI182	Treated				10	4			15
MI184	Treated	Hybrid	1			22		3	32
WI126	Treated					18			23
MI188	Untreated	Hybrid	1		7	31			19
MI194	Untreated				9			8	
MI195	Untreated				27			19	
MI196	Untreated	EWM	A	28			28		
MI200	Untreated				22			27	
MI201	Untreated	EWM	A	28			30		
MI121	Untreated				9	5		17	1
MI152	Untreated	EWM	A	16			20		
