# Supplementary material for: Hybrid watermilfoil lineages are more invasive and less sensitive to a commonly used herbicide than their exotic parent (Eurasian watermilfoil)
Source: Evol Appl. 2012 Nov 16;6(3):462–71. doi: 10.1111/eva.12027 (PMC3673474; doi:10.1111/eva.12027)
Supplement: Table S2 — Populations from the Lower Peninsula of Michigan, USA used in the 2,4-D sensitivity assays. [file eva0006-0462-sd4.pdf]

Table S2. Populations from the Lower Peninsula of Michigan, USA used in the 2,4-D sensitivity assays. Population identification, biotype identification used in 2,4-D sensitivity assays, and the genetic cluster from AFLPs in the minimum-spanning network (see Figs. 1 and 2). EWM = Eurasian watermilfoil

Population	2,4-D Assay Biotype ID	Genetic Cluster
MI102	hybrid	2
MI128	hybrid	2
MI133	hybrid	4
MI204	hybrid	2
MI233	hybrid	5
MI240	hybrid	2
MI101	EWM	B
MI116	EWM	A
MI134	EWM	B
MI137	EWM	A
MI140	EWM	A
MI147	EWM	A
MI156	EWM	A
MI169	EWM	A
MI173	EWM	A
