# Supplementary material for: Hybrid watermilfoil lineages are more invasive and less sensitive to a commonly used herbicide than their exotic parent (Eurasian watermilfoil)
Source: Evol Appl. 2012 Nov 16;6(3):462–71. doi: 10.1111/eva.12027 (PMC3673474; doi:10.1111/eva.12027)
Supplement: Table S3 — Responses of individual hybrid and Eurasian watermilfoil (EWM) populations from the Menominee River watershed to four treatments of 2,4-D and a control after 22 days of growth with (a) growth at a treatment of 2,4-D relative to growth at the control (Length treated/Length control), and (b) length gained. [file eva0006-0462-sd5.pdf]

Table S3. Responses of individual hybrid and Eurasian watermilfoil (EWM) populations from the Menominee River watershed to four treatments of 2,4-D and a control after 22 days of growth with a) growth at a treatment of 2,4-D relative to growth at the control (Length treatment / Length control) , and b) length gained. Both the mean and ± SEM (in parentheses) for each treatment are shown. Sample size is five individuals per treatment except at 100 µg/L for MI196 (N = 4) and at 500 µg/L for MI154 (N = 0)

a) Length $_{treated}$/Length $_{control}$	Eurasian Watermilfoil					
	MI196	MI152	MI201	MI154		
100 µg/L	0.79 (0.14)	0.92 (0.14)	0.78 (0.13)	0.85 (0.18)		
150 µg/L	0.34 (0.05)	0.56 (0.13)	0.68 (0.13)	0.50 (0.09)		
200 µg/L	0.34 (0.12)	0.54 (0.11)	0.44 (0.13)	0.22 (0.07)		
500 µg/L	0.19 (0.03)	0.09 (0.03)	0.25 (0.06)	NA		
	Hybrid					
	MI026	WI125	MI184	MI179	MI202	MI188
100 µg/L	0.73 (0.12)	0.83 (0.07)	0.95 (0.14)	1.09 (0.10)	1.05 (0.13)	1.07 (0.07)
150 µg/L	0.74 (0.10)	1.10 (0.11)	0.84 (0.14)	0.86 (0.11)	0.75 (0.15)	1.01 (0.12)
200 µg/L	0.71 (0.12)	0.88 (0.13)	0.79 (0.11)	0.72 (0.15)	0.79 (0.07)	1.01 (0.15)
500 µg/L	0.49 (0.09)	0.78 (0.14)	0.38 (0.10)	0.61 (0.17)	0.30 (0.04)	0.56 (0.14)
b) Length Gained (cm)	Eurasian Watermilfoil					
	MI196	MI152	MI201	MI154		
Control	30.56 (4.36)	18.52 (1.92)	27.68 (4.32)	22.18 (3.59)		
100 µg/L	24.05 (4.15)	17.00 (2.57)	21.48 (3.68)	18.76 (4.09)		
150 µg/L	10.54 (1.59)	10.40 (2.32)	18.80 (3.46)	11.02 (2.06)		
200 µg/L	10.44 (3.73)	10.04 (2.10)	12.26 (3.52)	4.92 (1.45)		
500 µg/L	5.92 (0.91)	1.66 (0.60)	6.88 (1.76)	NA		
	Hybrid					
	MI026	WI125	MI184	MI179	MI202	MI188
Control	51.24 (4.45)	49.94 (5.86)	46.46 (8.16)	47.84 (11.15)	41.6 (7.21)	35.56 (3.52)
100 µg/L	37.3 (6.38)	35.54 (2.84)	44.16 (6.73)	52.06 (4.95)	43.78 (5.28)	38.08 (2.57)
150 µg/L	37.76 (5.35)	47.06 (4.92)	39.00 (6.27)	41.06 (5.37)	31.3 (6.40)	35.82 (4.18)
200 µg/L	36.38 (6.34)	37.58 (5.58)	36.66 (5.04)	34.62 (6.95)	32.86 (2.87)	42.16 (6.21)
500 µg/L	25.08 (4.67)	33.46 (6.22)	17.62 (4.86)	29.24 (8.26)	12.46 (5.71)	19.86 (4.87)
