# Supplementary material for: Hybrid watermilfoil lineages are more invasive and less sensitive to a commonly used herbicide than their exotic parent (Eurasian watermilfoil)
Source: Evol Appl. 2012 Nov 16;6(3):462–71. doi: 10.1111/eva.12027 (PMC3673474; doi:10.1111/eva.12027)
Supplement: Table S4 — Responses of individual hybrid and Eurasian watermilfoil (EWM) populations from the Lower Peninsula of MI, USA to two treatments of 2,4-D and a control after 20 days of growth with (a) growth at a treatment of 2,4-D relative to growth at the control (Length treated/Length control), and (b) length gained (cm). [file eva0006-0462-sd6.pdf]

Table S4. Responses of individual hybrid and Eurasian watermilfoil (EWM) populations from the Lower Peninsula of MI, USA to two treatments of 2,4-D and a control after 20 days of growth with a) growth at a treatment of 2,4-D relative to growth at the control (Length treatment / Length control) , and b) length gained (cm). Both the mean and ± SEM (in parentheses) for each treatment are shown. Sample size is four individuals per treatment except for MI133 at 0 µg/L (N = 3) and MI233 (N=3 for all treatments)

| a) Length $_{treated}$/Length $_{control}$ | Eurasian Watermilfoil | | | | | | | | |
|---|---|---|---|---|---|---|---|---|---|
| | MI137 | MI101 | MI173 | MI140 | MI147 | MI156 | MI169 | MI134 | MI116 |
| 200 µg/L | 0.46 (0.06) | 0.75 (0.17) | 0.73 (0.23) | 0.55 (0.16) | 0.55 (0.10) | 0.94 (0.22) | 1.14 (0.21) | 0.68 (0.17) | 0.42 (0.04) |
| 500 µg/L | 0.19 (0.05) | 0.41 (0.19) | 0.43 (0.22) | 0.21 (0.05) | 0.17 (0.03) | 0.41 (0.14) | 0.79 (0.12) | 0.31 (0.10) | 0.28 (0.07) |
| | Hybrid | | | | | | | | |
| | MI128 | MI133 | MI240 | MI233 | MI204 | MI102 | | | |
| 200 µg/L | 0.79 (0.12) | 0.89 (0.18) | 1.48 (0.12) | 0.86 (0.07) | 0.80 (0.12) | 0.84 (0.09) | | | |
| 500 µg/L | 0.79 (0.19) | 0.74 (0.09) | 1.12 (0.22) | 0.90 (0.20) | 1.02 (0.10) | 0.82 (0.06) | | | |
| b) Length Gained (cm) | Eurasian Watermilfoil | | | | | | | | |
| | MI137 | MI101 | MI173 | MI140 | MI147 | MI156 | MI169 | MI134 | MI116 |
| Control | 30.13 (4.34) | 21.70 (8.65) | 25.60 (2.33) | 39.93 (3.13) | 45.35 (4.95) | 40.35 (7.81) | 22.20 (7.39) | 34.45 (2.51) | 37.70 (6.77) |
| 200 µg/L | 13.88 (1.74) | 16.23 (3.66) | 18.63 (5.81) | 21.95 (6.52) | 24.75 (4.42) | 29.28 (7.60) | 25.30 (4.62) | 23.55 (5.78) | 15.98 (1.48) |
| 500 µg/L | 5.83 (1.47) | 8.90 (4.09) | 11.03 (5.74) | 8.43 (1.95) | 7.50 (1.18) | 16.63 (5.52) | 17.55 (2.61) | 10.68 (3.44) | 10.50 (2.62) |
| | Hybrid | | | | | | | | |
| | MI128 | MI133 | MI240 | MI233 | MI204 | MI102 | | | |
| Control | 45.43 (13.39) | 61.03 (7.19) | 54.73 (7.69) | 49.77 (7.02) | 51.78 (10.56) | 40.15 (5.63) | | | |
| 200 µg/L | 36.05 (5.52) | 54.33 (11.13) | 60.55 (4.18) | 43.00 (3.68) | 41.58 (6.42) | 39.38 (7.02) | | | |
| 500 µg/L | 36.08 (8.46) | 45.00 (5.65) | 45.90 (9.05) | 44.00 (12.51) | 52.90 (5.39) | 33.03 (2.50) | | | |
